# Impact of Radiomics Parameters and Clinical Integration on Prognostication in Head and Neck Squamous Cell Carcinoma: A Multicenter Study

**DOI:** 10.3390/life16061027

**Published:** 2026-06-19

**Authors:** Hajar Moradmand, Jason Molitoris, Ranee Mehra, Lisa Schumaker, Erin Allor, Daria A. Gaykalova, Lei Ren

**Affiliations:** 1 Department of Radiation Oncology, University of Maryland School of Medicine, Baltimore, MD 21201, USA; jmolitoris@umm.edu; 2 Marlene and Stewart Greenebaum Comprehensive Cancer Center, University of Maryland School of Medicine, Baltimore, MD 21201, USA; ranee.mehra@umm.edu (R.M.);; 3 Institute for Genome Sciences, University of Maryland School of Medicine, Baltimore, MD 21201, USA; 4 Department of Otorhinolaryngology–Head and Neck Surgery, University of Maryland School of Medicine, Baltimore, MD 21201, USA; 5 Department of Oncology, Sidney Kimmel Comprehensive Cancer Center, Johns Hopkins University, Baltimore, MD 21201, USA

**Keywords:** radiomics, head and neck squamous cell carcinoma (HNSCC), feature stability, Cox survival analysis

## Abstract

Radiomics has the potential to improve risk stratification in head and neck squamous cell carcinoma (HNSCC), but clinical adoption is limited by inconsistent performance across institutions. A key source of variability is how radiomic features are generated, preprocessed, and selected prior to model development. This multicenter study evaluated how radiomics parameterization and feature selection strategies affect external model performance, feature stability, and time-to-event risk stratification. We studied pre-treatment CT scans from 752 patients with primary HNSCC from three hospitals. For each scan, 1648 radiomic features were computed using 20 different preparation methods that varied in scaling, outlier removal, and gray-level bin width. We compared five feature selection methods: Graph-FS with connected components, Boruta, Lasso, RFE-RF, and mRMR. The classification models used were Random Forest, XGBoost, CatBoost, and Logistic Regression. We measured performance using external ROC-AUC, bootstrap confidence intervals, Brier score, and RobustScore. Stability of feature selection was assessed using the Kuncheva and Jaccard indices. Cox proportional hazards models confirmed time-to-event results, and consensus SHAP analysis helped explain the models. Radiomics parameterization influenced model performance, and no single configuration was optimal across all analyses. Radiomics-only models outperformed clinical-only models, while clinical–radiomics models achieved the highest overall performance. mRMR and Lasso produced the highest average external AUCs, while Graph-FS showed the greatest stability. The best classification model achieved an external AUC of 0.817. In Cox validation, the best clinical–radiomics configuration achieved an external C-index of 0.662 and separated high- and low-risk patients in the external cohort.

## 1. Introduction

Radiomics refers to the high-throughput extraction of quantitative features from medical images, aiming to capture tumor heterogeneity, shape, and texture characteristics that may be imperceptible to the naked eye [1]. In head and neck squamous cell carcinoma (HNSCC), these imaging-derived features have been explored for predicting survival [2,3,4], treatment response [5], and recurrence risk [6]. Despite promising results, translation into routine clinical practice has remained challenging. Radiomics models are sensitive to differences in image acquisition, scanner characteristics, reconstruction algorithms, segmentation, preprocessing, and patient populations across institutions [7]. As a result, models that perform well in one setting may not generalize reliably to another [8].

This issue is especially relevant in HNSCC, which is not a single uniform disease. Prognosis differs according to tumor subsite, stage, treatment approach, smoking history, and HPV status [9,10]. The prognostic role of HPV is most established in oropharyngeal squamous cell carcinoma, although its effect may vary with smoking exposure, treatment approach, stage, and other clinical factors [11]. These sources of heterogeneity indicate that a single high-performing model is insufficient to demonstrate clinical usefulness. A clinically meaningful radiomics pipeline should show consistent performance across centers, parameter settings, and clinical contexts, particularly when key prognostic variables are not uniformly available [8].

Feature selection plays an important role in addressing these limitations by reducing dimensionality, minimizing redundancy, and improving model interpretability [12,13]. Commonly used approaches include Minimum Redundancy Maximum Relevance (mRMR) [14], the Least Absolute Shrinkage and Selection Operator (Lasso) regression [15], Recursive Feature Elimination (RFE) with Random Forest [16], and Boruta [17]. However, these methods do not always select stable feature subsets when applied across different folds, preprocessing settings, or institutions. This instability is a major concern in HNSCC radiomics, where both imaging protocols and patient characteristics can vary substantially [8,18].

Graph-based feature selection (Graph-FS) offers an alternative approach to addressing this problem. Instead of evaluating features independently, Graph-FS represents radiomic features as nodes in a graph, where edges capture statistical relationships between features [19,20,21]. In the connected-components approach, related features are grouped, and a representative feature is selected from each group. This may reduce redundancy while preserving complementary information, which could improve feature selection stability across heterogeneous datasets [22].

Another important source of variability is the way radiomic features are generated. Choices such as normalization scale, outlier removal, and gray-level bin width can change feature values even when the same tumor volume is analyzed. Our previous work in glioblastoma imaging also showed that preprocessing decisions can substantially affect feature stability across imaging modalities [23]. In HNSCC, however, the downstream effects of these parameter choices on feature-selection stability and external model performance remain insufficiently studied.

Post hoc harmonization approaches, such as ComBat, are often proposed to reduce center-related differences in radiomics data [24]. These methods can help adjust some distributional differences across institutions. However, they cannot fully correct the variability introduced earlier during feature extraction, as different preprocessing or discretization settings yield distinct feature representations. For this reason, radiomics parameterization should be evaluated directly rather than treated as a technical detail. A parameter-aware evaluation framework is needed to identify settings that are not only predictive but also stable and reproducible across centers.

Clinical variables are also essential when assessing the value of radiomics. Age, AJCC stage, T-category, and N-category remain important prognostic factors in HNSCC and provide a clinical context for imaging features [22,23].

In this study, we examined how choices in radiomics settings and feature selection methods affect survival prediction in a cohort of patients with HNSCC from multiple centers. Instead of focusing on a single best model, we wanted to identify modeling approaches that perform well across different centers and data processing methods. We compared Graph-FS with common feature selection methods across various radiomics settings, classifiers, and ways of combining clinical data. We checked model performance using tests on new data, time-to-event analysis, and measures of feature consistency. To better summarize the models’ reliability, we used RobustScore, which penalizes variability, along with a consensus SHAP analysis. Our goal was to create a radiomics modeling approach that is accurate, reliable, and easy to understand in clinical settings across diverse multicenter data.

## 2. Methods

### 2.1. Study Design and Cohort

**Figure 1:**
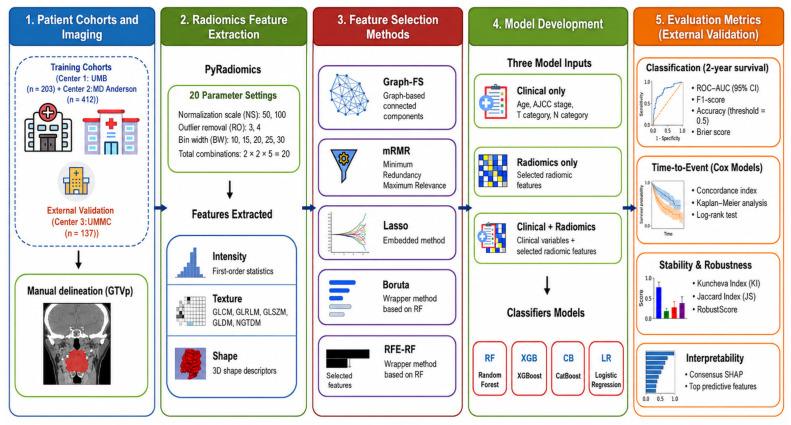
Study workflow. Patients with HNSCC underwent pre-treatment CT imaging. The primary gross tumor volume (GTVp) was delineated and used to extract radiomics features. Radiomics features were extracted across multiple preprocessing parameter settings. Feature selection was performed using Graph-FS and standard feature selection methods. Selected features were used to train radiomics-only and clinical–radiomics models using CatBoost, Random Forest, XGBoost, and Logistic Regression. Clinical-only models were trained separately as reference baselines. External validation was performed in an independent cohort using ROC-AUC, Brier score, feature selection stability metrics, RobustScore, and Cox time-to-event analysis.The training cohorts included patients from the University of Maryland, Baltimore cohort (UMB; *n* = 203; 2006–2017) and the MD Anderson Cancer Center cohort (MDACC; *n* = 412; 2003–2013; publicly available through The Cancer Imaging Archive) [25]. Patients from the University of Maastricht Medical Center cohort (UMMC; *n* = 137) were used as an independent external validation cohort [1,26].

This multicenter retrospective study included 752 patients with primary head and neck squamous cell carcinoma (HNSCC) from three institutions. The overall study workflow is shown in Figure 1.

The cohorts differed in clinical composition. Treatment also varied across cohorts and reflected institutional practice patterns, including surgery alone, definitive radiotherapy, adjuvant radiotherapy, and chemoradiotherapy. Baseline demographic and clinical characteristics are summarized in Table 1.

The primary binary endpoint was 2-year survival. Patients who survived beyond 2 years were labeled as alive at 2 years, and patients who died within 2 years were labeled as deceased at 2 years. Patients censored before 2 years were excluded from the binary classification analysis when their 2-year survival status could not be determined. Time-to-event survival was also evaluated using Cox proportional hazards models, as described below.

To assess differences among cohorts, Welch’s ANOVA was used for continuous variables (e.g., age), and Chi-square tests were used for categorical variables (e.g., sex, T-category, N-category, AJCC stage, and 2-year survival). A *p*-value < 0.05 was considered statistically significant.

### 2.2. Image Preprocessing and Radiomics Feature Extraction

Expert readers contoured the gross tumor volumes or used verified clinical delineations when available. Radiomic features were extracted from pre-treatment CT scans using PyRadiomics, following IBSI-compliant principles [26,27]. Features were extracted from the GTVp region.

To evaluate the impact of preprocessing parameterization, we generated 20 radiomics parameter configurations. These configurations were based on combinations of the normalization scale (NS ∈ {50, 100}), the outlier removal threshold (RO ∈ {3, 4}), and the gray-level bin width (BW ∈ {10, 15, 20, 25, 30}). Each parameter setting produced a separate radiomics feature matrix. Full PyRadiomics extraction settings are provided in Supplementary Table S1.

### 2.3. Clinical Variables and Model Groups

The available clinical variables included age, AJCC stage, T-Category, and N-Category. AJCC stage, T-Category, and N-Category were treated as categorical variables rather than continuous variables because the distance between categories is not assumed to be linear.

Three model groups were evaluated. The clinical-only model used age, AJCC stage, T-category, and N-category and served as a reference baseline. The radiomics-only model used only selected radiomic features. The clinical–radiomics model combined selected radiomic features with the available clinical variables. This design allowed us to assess the prognostic value of radiomics alone and the incremental contribution of clinical variables when combined with radiomics.

This comparison allowed us to assess whether the radiomics profile had prognostic value on its own and whether it added useful information beyond standard clinical predictors [28].

### 2.4. Feature Selection (FS) Methods

We compared five feature selection strategies: Graph-FS using connected components, Boruta, Lasso, Recursive Feature Elimination with Random Forests, and mRMR. Detailed mathematical descriptions and implementation details are provided in the Supplementary Information, including a schematic of the Graph-FS workflow (Supplementary Figure S1).

Graph-Based Feature Selection (Graph-FS) represented radiomic features as nodes in a feature-similarity graph, with edges indicating pairwise associations between features. Connected components were used to identify groups of related features, and a representative feature was retained from each component. This approach was intended to reduce redundancy while preserving complementary feature information [29,30,31].

Boruta was used as a random forest–based wrapper method that compares feature importance against permuted shadow features to identify relevant variables [17].

Least Absolute Shrinkage and Selection Operator (Lasso) was used as an embedded method that applies ℓ1-regularized regression to induce sparsity and shrink uninformative coefficients to zero [15].

Recursive Feature Elimination with Random Forests (RFE-RF) was used as a wrapper method that recursively removes the least important features based on random forest importance scores [16].

Minimum Redundancy Maximum Relevance (mRMR) was used as a filter-based method that selects features by maximizing mutual information with the outcome while minimizing redundancy among selected features [14].

All feature selection steps were applied independently for each radiomics parameterization using the training cohorts. The selected features were then applied to the external validation cohort.

### 2.5. Feature Selection Stability Analysis

Feature selection stability was assessed using the Kuncheva and Jaccard indices. These metrics were used to quantify how consistently each method selected similar feature subsets across parameter settings and cohorts.

The Kuncheva Index adjusts observed overlap between two feature subsets for the overlap expected by chance [32]. Let $S_{i}$ and $S_{j}$ denote two feature subsets of size *k*, selected from a feature space of size *d*. If ${r=|S}_{i}\cap S_{j}|$ is the number of shared features, the Kuncheva index (*KI*) is defined in Equation (1):

(1) $${KI(S}_{i},S_{j})=\frac{rd-k^{2}}{k(d-k)}$$

The Kuncheva index ranges from −1 to 1. A value of 1 indicates identical subsets and perfect stability. A value near 0 indicates that overlap is like random selection, and negative values indicate that overlap is less than expected by chance.

The Jaccard Index (also called Jaccard Similarity coefficient, JSC) measures the similarity between two selected feature subsets. It is calculated as shown in Equation (2), where *x* and *y* represent two feature sets, and $\left|x_{i}\cap y_{j}\right|$ is the number of shared features and $\left|x_{i}\cup y_{j}\right|$ is the total number of unique features in both sets [33].

(2) $$J\left(x_{i},y_{j}\right)=\frac{\left|x_{i}\cap y_{j}\right|\;}{\left|x_{i}\cup y_{j}\right|}=\frac{\left|x_{i}\cap y_{j}\right|}{\left|x_{i}\right|+\left|y_{j}\right|-\left|x_{i}\cap y_{j}\right|}$$

The Jaccard Index ranges from 0, indicating no overlap, to 1, indicating identical sets. To assess overall stability, we averaged the Jaccard Index across all pairs to selected feature subsets *P* (Equation (3)):

(3) $$JS=\frac{2}{\left|P\right|\times (\left|P\right|-1)}\sum_{i=1}^{\left|P\right|-1}\sum_{j=i+1}^{\left|P\right|}J\left(x_{i},y_{j}\right)$$

For each method, pairwise Kuncheva and Jaccard values were calculated across different cross-validation folds and parameter settings. These values were then averaged to obtain an overall stability score for each method.

### 2.6. Robustness and Reproducibility Ranking

To compare methods across many parameter settings, we used a RobustScore (Equation (4)) that considered both average model performance and performance variability:

(4) RobustScore=mean(AUC)−λ.std(AUC)

The purpose of this score was to avoid ranking a method highly only because it achieved a strong result in a small number of settings. A method with a high mean AUC but large variation across parameterizations was penalized, whereas a method with consistently good performance received a higher score. In this way, RobustScore reflects both discrimination and reproducibility [8,34,35]. The primary analysis used λ=0.5, representing a moderate penalty for variability. To assess whether the ranking depended on this choice, sensitivity analyses were also performed using λ=0.25, 0.50, and 0.75.

### 2.7. Learning Pipeline, Class Imbalance Handling, and Tuning

For radiomics-based models, feature selection was performed separately for each radiomics parameter setting. The selected radiomic features were then used to train radiomics-only and clinical–radiomics models. Clinical-only models were trained separately using only the available clinical variables. Four classifiers were evaluated: Random Forest, XGBoost, CatBoost, and Logistic Regression [36,37,38].

All preprocessing steps were kept within the modeling pipeline. Continuous variables were imputed with the median. Categorical variables, including AJCC stage, T-category, and N-category, were imputed with the most frequent category and converted into indicator variables.

Because the 2-year survival endpoint was imbalanced, class weights were applied during model fitting. This approach increased the penalty for misclassifying the minority class without generating synthetic samples. Synthetic oversampling was not used in the final analysis to reduce the risk of introducing artificial patterns in the high-dimensional radiomics feature space. Hyperparameter tuning was performed in the combined Center 1 and Center 2 training cohort. For each classifier, grid search with 3-fold cross-validation was used to identify the hyperparameters that maximized ROC-AUC. After tuning, each final model was refit on the full Center 1 and Center 2 training data and evaluated once on the independent Center 3 external validation cohort.

The final classification analysis included 20 radiomics parameterizations, 5 feature selection methods, 4 classifiers, and 2 radiomics-based model groups, resulting in 800 radiomics-based classification models. Four additional clinical-only models were trained as reference baselines, one for each classifier.

### 2.8. External Validation and Evaluation Metrics

After tuning, we applied the optimal hyperparameters to the resampled training set and evaluated performance on the external test set (Center 3). The primary discrimination metric was ROC–AUC, with 95% confidence intervals estimated by nonparametric bootstrap with 1000 resamples [39,40]. We also reported F1-score, accuracy at a 0.5 probability threshold, and Brier score [41]. The Brier score was used to assess the accuracy of predicted probabilities, with lower values indicating better probabilistic performance.

As we used many model configurations, results were summarized using mean, median, standard deviation, confidence intervals, RobustScore, and the proportion of models exceeding clinically relevant AUC thresholds. 

### 2.9. Time-to-Event Survival Analysis

Because binary 2-year survival does not use the full follow-up time, we performed an additional time-to-event analysis using Cox proportional hazards models. Cox models were trained on Centers 1 and 2 and externally validated on Center 3. Three predictor groups were evaluated: clinical-only, radiomics-only, and combined clinical–radiomics models. For radiomics-only and combined clinical–radiomics models, each feature selection method was evaluated independently for each radiomics parameterization. Clinical-only models were trained separately using the same classifiers and validation strategy, but without radiomics parameterization or feature selection because no radiomic features were included.

To reduce overfitting, the number of radiomic predictors included in each Cox model was limited to the top-selected features available for that parameterization and method. Continuous predictors were standardized using the training data, and categorical clinical variables were one-hot encoded.

Model discrimination was evaluated using the concordance index on the external validation cohort. Predicted risk scores were used to stratify external validation patients into high- and low-risk groups based on the median risk score. Kaplan–Meier curves were generated for the two risk groups, and the log-rank test was used to compare survival distributions.

### 2.10. Software

All experiments were conducted on a Windows 11 Pro workstation with an Intel Core i9-14900K CPU (32 cores) (Santa Clara, CA, USA) and an NVIDIA GeForce RTX 4070 GPU (Santa Clara, CA, USA). Python 3.11 was used for preprocessing and feature extraction. SimpleITK (v2.3.1) performed image processing and isotropic resampling, and PyRadiomics (v3.1.0) extracted first-order, texture, and shape features from pre-treatment CT scans.

Feature selection was performed using Boruta, Lasso, RFE, mRMR, and Graph-FS, implemented with scikit-learn (v1.3.0), mRMR-selection (v0.2.8), and networkx (v3.1). Stability and reproducibility were assessed using the Jaccard Index, Dice Similarity Index, and Kendall’s W, calculated with Python set operations, stats models (v0.14.0), and pingouin (v0.5.3).

Ensemble models, primarily XGBoost (v3.0.2) and CatBoost (v1.2.8), were used for classification. These models were accessed through their native Python APIs and integrated with the scikit-learn framework.

## 3. Results

For the radiomics-based analysis, we evaluated 800 classification models across 20 radiomics parameterizations, five feature selection methods, four classifiers, and two model groups: radiomics-only and clinical–radiomics. Clinical-only models were trained separately as reference baselines because they did not depend on radiomics parameterization or feature selection.

The highest individual external AUC was 0.817. However, because many model configurations were tested, the results are presented mainly as aggregate performance distributions, parameter-level trends, and stability metrics rather than a single best-performing model.

### 3.1. Impact of Radiomics Parameterization

Radiomics parameterization affected external model performance. Normalization scale, outlier removal, and bin width all influenced AUC values, and no single parameter setting was uniformly optimal across all methods and classifiers.

**Figure 2:**
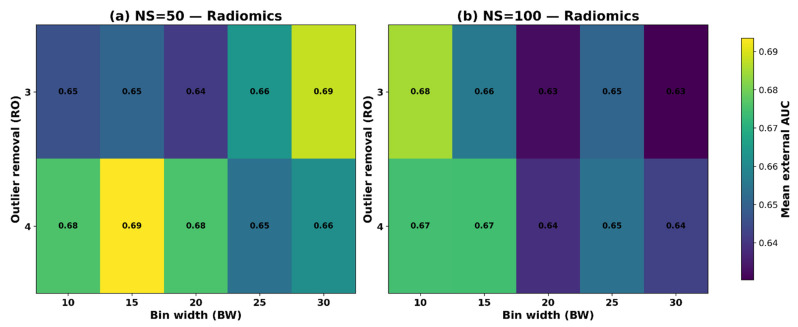
Impact of radiomics parameterization on radiomics-only model performance. Heatmaps show the mean external AUC across outlier-removal and bin-width combinations for radiomics-only models, stratified by normalization scale: (**a**) NS = 50 and (**b**) NS = 100. Values represent the average external AUC across feature selection methods and classifiers.

For radiomics-only models, the effect of parameterization is shown in Figure 2. Models extracted with NS = 50 performed better on average than those extracted with NS = 100, with mean external AUCs of 0.689 and 0.663, respectively. The highest radiomics-only values were observed for NS = 50 at RO = 3/BW = 30 and RO = 4/BW = 15, both reaching approximately 0.72. For NS = 100, the best values were observed at RO = 4/BW = 10 and RO = 4/BW = 15, both reaching approximately 0.69.

A similar trend was observed after adding clinical variables. Clinical–radiomics models had a higher mean AUC with NS = 50 than with NS = 100, with mean AUCs of 0.708 and 0.695, respectively. Outlier removal also influenced performance, with mean AUC increasing from 0.667 to 0.685 in radiomics-only models and from 0.694 to 0.709 in clinical–radiomics models when RO increased from 3 to 4.

The effect of bin width was less direct. In radiomics-only models, BW = 15 and BW = 10 showed the highest mean AUCs, followed closely by BW = 30. In clinical–radiomics models, BW = 10 had the highest mean AUC, followed by BW = 30 and BW = 15. The strongest robust clinical–radiomics parameter setting was Rad_ns50_ro4_bw15, with a mean AUC of 0.735 and a robust AUC score of 0.709. For radiomics-only models, the strongest robust setting was Rad_ns50_ro3_bw30, with a mean AUC of 0.718 and a robust AUC score of 0.696.

### 3.2. Clinical Contribution and Incremental Value of Radiomics

**Figure 3:**
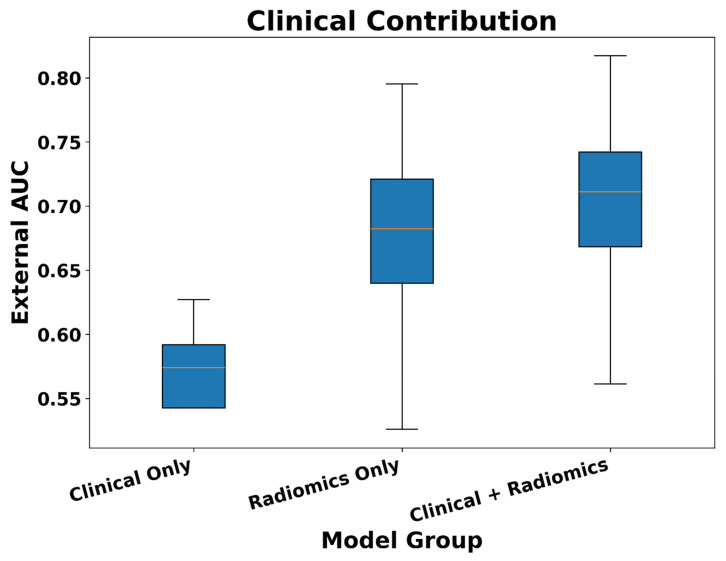
Clinical contribution and incremental value of radiomics. Boxplots show the distributions of external AUCs for the clinical-only, radiomics-only, and clinical–radiomics models.

The aggregate performance of the clinical-only, radiomics-only, and clinical–radiomics models is summarized in Table 2 and illustrated in Figure 3. Clinical-only models were included as reference baselines and achieved a mean external AUC of 0.560 and a median AUC of 0.574. Radiomics-only models showed higher discrimination, with a mean AUC of 0.676 and a median AUC of 0.682. The combined clinical–radiomics models had the highest aggregate performance, with a mean AUC of 0.702 and a median AUC of 0.711.

Adding clinical variables to radiomics increased the mean AUC from 0.676 to 0.702. Although the improvement was modest, it was consistent across the overall analysis. Accuracy and F1-score showed a different pattern: clinical-only models exhibited higher threshold-dependent performance than radiomics-only models. Because AUC was the primary discrimination metric, the main finding was that radiomics improved external risk discrimination relative to the available clinical-only baseline, while the combined clinical–radiomics models achieved the strongest overall discrimination.

### 3.3. External Validation Across Feature Selection Methods

**Figure 4:**
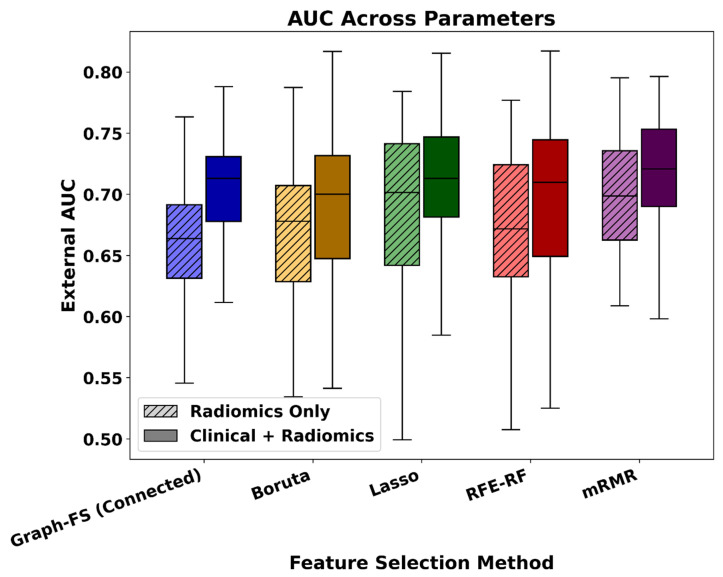
External AUC distributions across feature selection methods. Boxplots show the distributions of external AUCs across radiomics parameter settings and classifiers for each feature selection method. Radiomics-only and clinical–radiomics models are shown side by side.

External validation performance across feature selection methods is summarized in Table 3 and Figure 4. In radiomics-only models, mRMR achieved the highest mean external AUC, followed by Lasso. The mean AUC was 0.697 for mRMR, 0.692 for Lasso, 0.667 for RFE-RF, 0.666 for Boruta, and 0.658 for Graph-FS.

After adding clinical variables, the mean AUC increased for all feature selection methods. In the clinical–radiomics models, mRMR again showed the highest mean AUC at 0.715, followed by Lasso at 0.707 and Graph-FS at 0.703. RFE-RF and Boruta had mean AUCs of 0.692 and 0.691, respectively.

As shown in Figure 4, the AUC distributions overlapped across methods, indicating that the differences among the top-performing methods were modest. Although Graph-FS did not have the highest mean AUC, it remained close to mRMR and Lasso in the clinical–radiomics setting. Graph-FS also had the highest proportion of clinical–radiomics models with AUC ≥ 0.70, reaching this threshold in 62.5% of models, compared with 61.3% for Lasso and mRMR, 53.8% for RFE-RF, and 48.8% for Boruta.

### 3.4. Robustness and Feature Selection Stability

**Figure 5:**
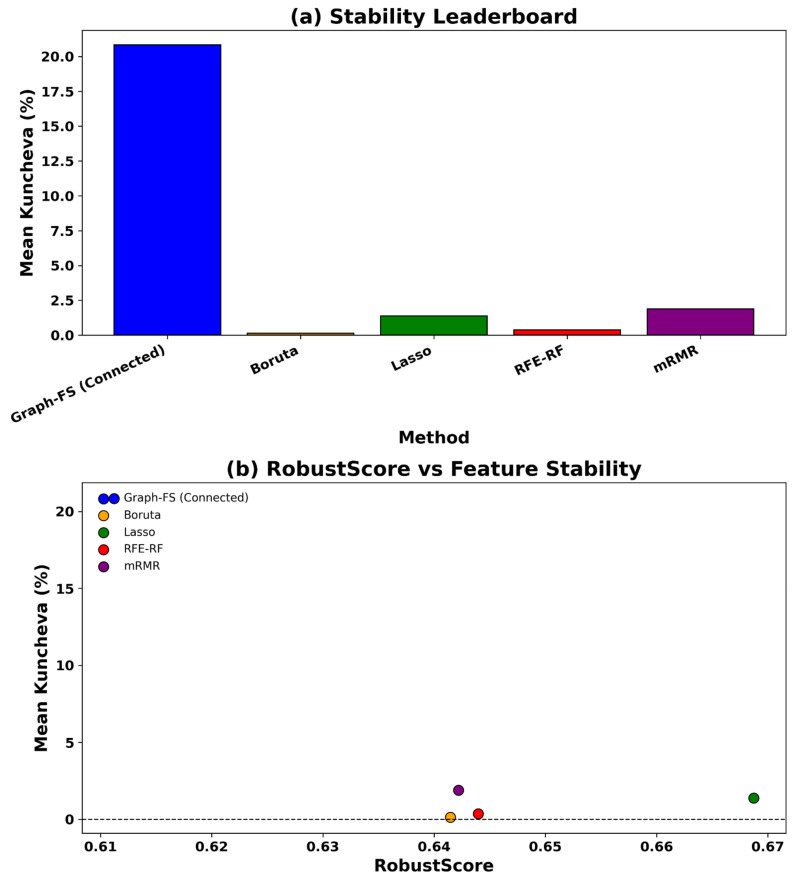
Feature-selection stability. (**a**) Overall mean Kuncheva index across feature selection methods. (**b**) Relationship between RobustScore and the overall mean Kuncheva index. Higher Kuncheva values indicate greater reproducibility of selected feature subsets.

Feature-selection stability is summarized in Figure 5. Graph-FS showed the highest overall mean Kuncheva index, indicating the most reproducible feature selection across parameter settings. The overall mean Kuncheva value was 18.5% for Graph-FS, compared with 3.9% for mRMR, 1.4% for Lasso, 0.5% for RFE-RF, and 0.5% for Boruta.

The RobustScore–stability plot showed a similar pattern. Graph-FS had the highest Kuncheva stability, while mRMR had the highest RobustScore. The remaining conventional methods clustered near low Kuncheva values, indicating limited overlap among selected feature subsets.

Feature selection stability can depend on the number of features chosen. We summarized the number of features selected by each method. In our main analysis, Graph-FS selected fewer features on average than the other methods (mean ± SD: 8.4 ± 4.8; median: 8; IQR: 4–12; range: 2–18). In contrast, Boruta, Lasso, RFE-RF, and mRMR each selected 10 features for every configuration (see Supplementary Table S3). This difference may account for Graph-FS’s higher stability. To assess this, we conducted a fixed-size sensitivity analysis, limiting all methods to 10 features per configuration. Even under this constraint, Graph-FS remained the most stable, indicating its stability is not solely due to selecting fewer features (see Supplementary Table S4 and Supplementary Figure S3).

RobustScore sensitivity analysis showed that the overall ranking was not driven by the selected penalty weight. (Supplementary Table S2) Across *λ* = 0.25, 0.50, and 0.75, the relative pattern remained consistent, with methods showing higher mean AUC and lower variability maintaining higher RobustScore values. The primary results are reported using *λ* = 0.5.

### 3.5. Time-to-Event External Validation

**Figure 6:**
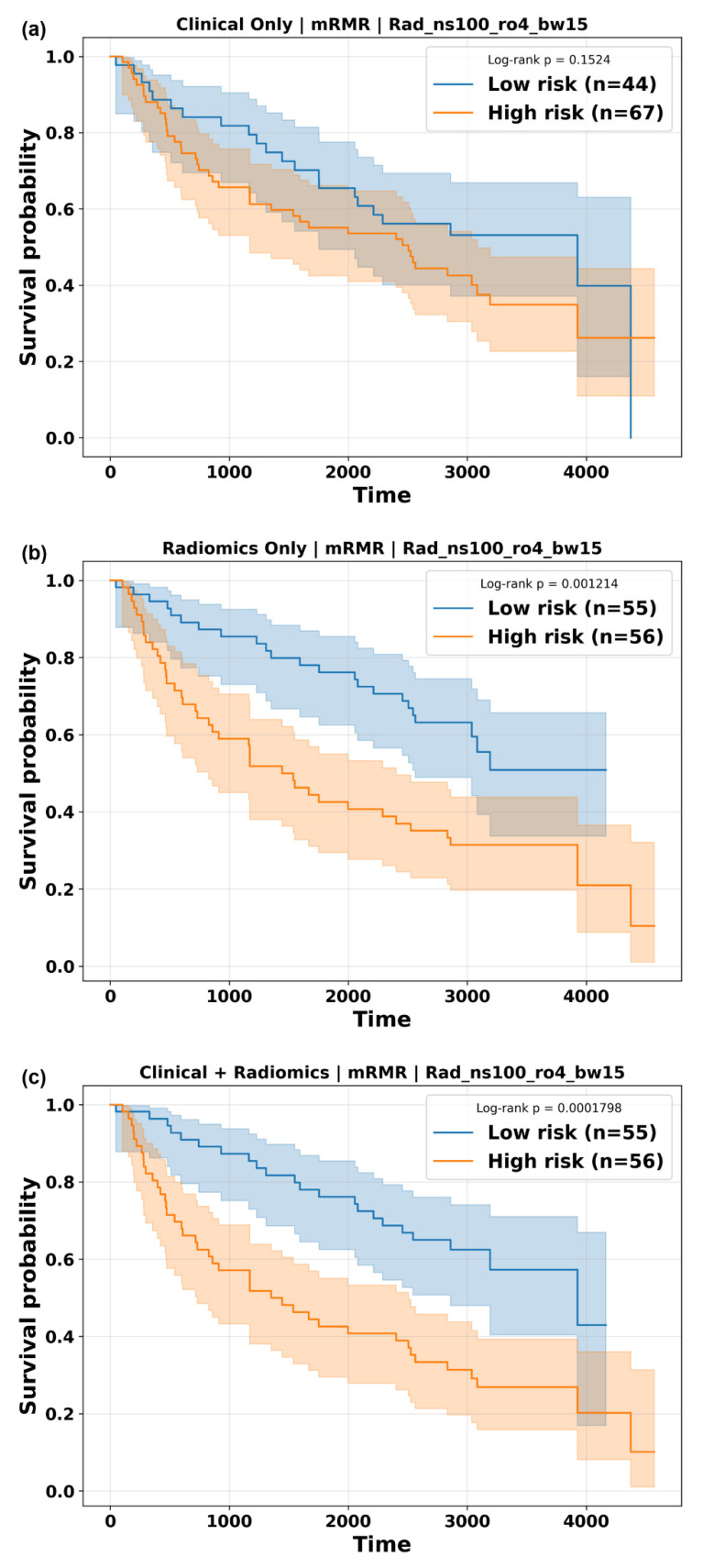
Time-to-event external validation. Kaplan–Meier curves for the external validation cohort using Cox risk scores from (**a**) clinical-only, (**b**) radiomics-only, and (**c**) clinical–radiomics models. Risk groups were defined using the median predicted Cox risk score. The clinical–radiomics model shown corresponds to the best individual Cox configuration, mRMR with Rad_ns100_ro4_bw15.

Cox proportional hazards model performance across parameter settings and feature selection methods is summarized in Table 4 and representative Kaplan–Meier curves are shown in Figure 6.

The clinical-only Cox baseline achieved an external C-index of 0.567 and did not significantly separate high- and low-risk groups in the external cohort (Figure 6a; log-rank *p* = 0.1524). Among radiomics-only Cox models, Lasso achieved the highest mean external C-index of 0.583. The representative radiomics-only model showed significant separation between risk groups (Figure 6b; log-rank *p* = 0.0012).

Clinical–radiomics Cox models showed higher aggregate time-to-event performance than radiomics-only models. The highest mean external C-index was observed for Lasso clinical–radiomics models at 0.598, followed by Boruta at 0.592, mRMR at 0.591, Graph-FS at 0.591, and RFE-RF at 0.587. The best individual Cox configuration was the mRMR clinical–radiomics model using Rad_ns100_ro4_bw15, which achieved an external C-index of 0.662 and separated 55 low-risk and 56 high-risk patients in the external cohort (Figure 6c; log-rank *p* = 0.00018).

### 3.6. Consensus SHAP Analysis

**Figure 7:**
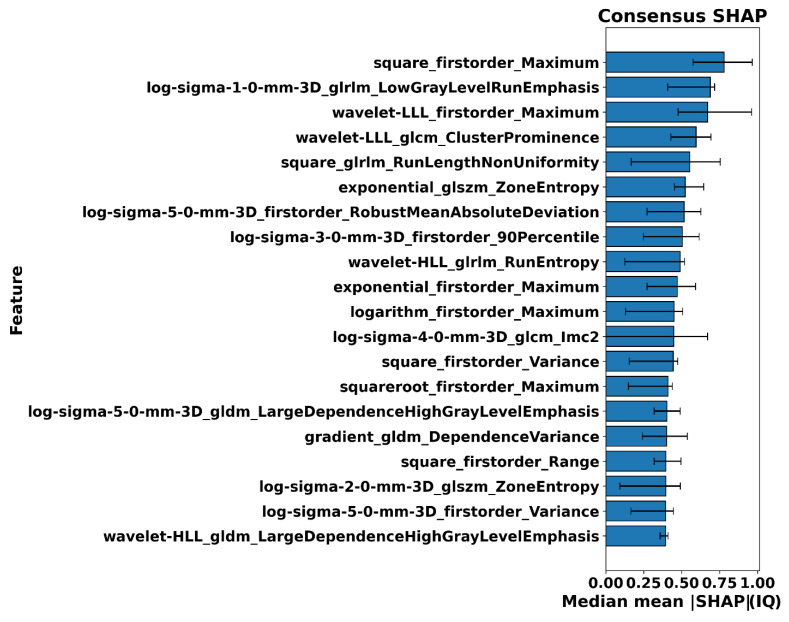
Consensus SHAP feature importance. The top 20 features are ranked by median mean absolute SHAP value across model configurations. Error bars represent the interquartile range.

Consensus SHAP analysis was performed as an exploratory interpretability analysis and is shown in Figure 7. The highest-ranked features were mainly first-order intensity and texture features from transformed images. The top features included square_firstorder_Maximum, wavelet-LLL_firstorder_Maximum, log-sigma-1-0-mm-3D_glrlm_LowGrayLevelRunEmphasis, wavelet-LLL_glcm_ClusterProminence, square_glrlm_RunLengthNonUniformity, and exponential_glszm_ZoneEntropy.

These results indicate that model predictions were influenced by both intensity distribution and texture heterogeneity. SHAP was used only to describe model behavior. These features were not interpreted as validated biological biomarkers.

## 4. Discussion

In this multicenter study, we examined how radiomics parameter settings and feature selection methods affect survival prediction in HNSCC. One reason for doing this work was that a radiomics model can appear strong when only a single optimized setting is reported, even though its performance may vary with preprocessing, feature selection, or clinical variables. To avoid relying on a single favorable result, we evaluated multiple parameter settings and summarized the findings using external validation, RobustScore, feature-selection stability, and Cox survival analysis.

Radiomics settings affected model performance. The clearest differences were seen for normalization scale and outlier removal. Models generated with NS = 50 generally performed better than those generated with NS = 100, and RO = 4 gave higher average AUC values than RO = 3. The effect of bin width was less consistent, suggesting that it may depend on other preprocessing choices in the pipeline. These findings show that radiomics parameter settings should be tested during model development, especially when the model is intended to work across different institutions.

Our results also show why reporting only the best-performing model can be misleading. Some model configurations reached relatively high external AUC values, but the average performance across all tested pipelines was lower. For this reason, we used RobustScore to combine average AUC with variability across parameter settings. This approach helped identify models that were not only predictive but also less sensitive to changes in the radiomics workflow.

The feature selection methods showed different strengths. mRMR and Lasso achieved the highest average external AUCs, suggesting that these established methods remain strong for prediction. Graph-FS, however, selected the most stable feature subsets across parameter settings, based on Kuncheva and Jaccard analyses. This distinction is important because a feature signature that changes substantially across settings is difficult to interpret and reproduce, even if its prediction performance is acceptable. In this study, Graph-FS was not the top method in terms of average AUC, but it was the strongest method for feature-selection stability.

We also examined whether the stability advantage of Graph-FS was related to the number of selected features. In the main analysis, Graph-FS selected slightly fewer features on average than the conventional methods. This may partly explain its higher stability, as a smaller group of representatives with fewer redundant features may be more consistent across parameter settings. It may also partly explain why Graph-FS had a lower average AUC than mRMR and Lasso, since a smaller feature set may leave out some predictive information. To check this, we performed a fixed-size sensitivity analysis in which all methods were constrained to retain 10 features per configuration. Graph-FS still showed the highest stability in this setting, suggesting that its stability advantage was not solely attributable to selecting fewer features. It likely also reflects how Graph-FS groups correlated features and retains representative features from connected components.

The multicenter design introduced substantial clinical and imaging heterogeneity. This is useful because it provides a realistic external validation setting, but it also makes model generalization more difficult. HNSCC includes tumors from different anatomical sites, including the oral cavity and oropharynx, with differences in prognosis, treatment, HPV status, smoking association, and imaging appearance. The limited overlap in selected features across centers likely reflects this heterogeneity. Therefore, the stability advantage of Graph-FS should be interpreted as improved consistency across parameter settings, not as complete protection against differences between centers.

Models that combined clinical and radiomics data achieved the best overall performance. This suggests that radiomics added prognostic information beyond the clinical variables available in this study, while clinical factors still provided important context. However, the clinical-only model should be viewed as a reference baseline, not a complete clinical prognostic model. It included only variables consistently available across cohorts: age, AJCC stage, T-category, and N-category. Important factors such as HPV status, smoking history, detailed tumor subsite, and treatment information were not available for all patients. Therefore, our findings do not show that radiomics is superior to a complete clinical model. Rather, they show that CT-based radiomics provided additional prognostic information beyond the limited clinical baseline available in this dataset.

The Cox survival analysis added a time-to-event perspective beyond the binary 2-year survival endpoint. Clinical–radiomics Cox models had higher external C-index values than clinical-only or radiomics-only models and more often separated high- and low-risk groups in Kaplan–Meier analysis. The best combined Cox model showed clear risk-group separation in the external validation cohort. These results suggest that the combined imaging-clinical scores captured information related to survival time, not only 2-year outcome status. Still, the C-index values were moderate, which is expected given the differences among cohorts and the absence of several important clinical variables.

Consensus SHAP analysis was used to explore model behavior. The most influential features were mainly first-order intensity and texture features from transformed images. This suggests that model predictions were influenced by intensity distribution and spatial heterogeneity within the tumor. These findings are consistent with the idea that radiomics can capture quantitative tumor information that may not be fully described by visual assessment alone. However, SHAP values explain model output and should not be treated as biological validation. Future studies should evaluate these features together with pathology, HPV status, history of smoking, treatment response, and molecular markers before considering them clinically actionable.

This study has several limitations. First, the cohorts differed in clinical composition, tumor subsite, stage distribution, treatment approach, imaging protocol, and outcome distribution. This reflects real-world multicenter data, but it may also introduce confounding. Second, HPV status and smoking history were not available for all centers, which is especially important in HNSCC and particularly in oropharyngeal cancer. Third, detailed treatment information and tumor subsite were not consistently available, which limited the clinical-only baseline. Fourth, although we used external validation, additional independent cohorts are needed before clinical implementation.

Another limitation is the large number of model configurations. Testing many configurations was necessary to study the effects of radiomics parameterization and feature selection, but it also increased the risk of overemphasizing a few favorable results. To reduce this concern, we emphasized aggregate performance, RobustScore, feature-selection stability, and time-to-event validation rather than only the highest AUC.

ComBat harmonization was not used as the primary strategy in this study. Harmonization can reduce scanner- or institution-related differences, but it does not fully address variability introduced earlier during feature extraction. Variability from radiomics parameter choices and variability from center differences are related but distinct. Future work should study how harmonization, parameter-aware feature extraction, and stable feature selection can be combined in larger multicenter datasets.

In summary, these findings suggest that radiomics can provide useful prognostic information in multicenter HNSCC, especially when combined with clinical data. However, radiomics pipelines should not be judged only by their best-performing configuration. Feature reproducibility, robustness across parameter settings, and time-to-event validation are also important when evaluating whether a model is likely to generalize beyond the development cohort.

## 5. Conclusions

Radiomics settings and feature selection choices affected survival prediction in this multicenter HNSCC cohort. Radiomics-only models outperformed the available clinical-only baseline, and the best overall results were achieved when radiomics and clinical variables were combined.

The results also showed that prediction performance and feature stability are not the same. mRMR and Lasso gave the highest average AUCs, while Graph-FS selected features more consistently across parameter settings. This is important because a model with a high AUC may still rely on features that are difficult to reproduce.

These findings support a more careful way of evaluating radiomics models. Instead of focusing only on the highest AUC, future studies should also consider stability, robustness across parameter settings, and time-to-event validation. Further validation in larger cohorts with complete HPV status, smoking history, treatment details, and tumor subsite data is needed to clarify the clinical role of radiomics-based risk stratification in HNSCC.

## Figures and Tables

**Table 1:** Baseline Demographics and Clinical Characteristics.

Variable	Category	MD Anderson (*n* = 412)	UMB (*n* = 203)	UMMC (*n* = 137)	*p*-Value
**Age (years)**	Median (range)	58 (29–80)	61 (28–97)	62 (44–83)	1.45 × 10^−5^
**Gender**	Male	86%	65.5%	81.5%	5.56 × 10^−9^
	Female	14%	34.5%	18.5%	
**2-yr Survival**	Alive	377 (91.5%)	113 (55.6%)	107 (78%)	5.1 × 10^−48^
	Deceased	35 (8.5%)	90 (44.4%)	30 (22%)	
**AJCC Stage**	I	3	45	24	7.08 × 10^−26^
	II	13	29	11	
	III	57	36	23	
	IV	339	93	79	
**T-Category**	T1	87 (21.1%)	56 (27.6%)	35 (25.5%)	2.33 × 10^−8^
	T2	170 (41.3%)	54 (26.6%)	32 (23.4%)	
	T3	93 (22.6%)	29 (14.3%)	24 (17.5%)	
	T4	62 (15.0%)	64 (31.5%)	46 (33.6%)	
**N-Category**	N0	37 (9.0%)	103 (50.7%)	60 (43.8%)	8.46 × 10^−37^
	N1	45 (10.9%)	31 (15.3%)	16 (11.7%)	
	N2	318 (77.2%)	55 (27.1%)	58 (42.3%)	
	N3	12 (2.9%)	14 (6.9%)	3 (2.2%)	

**Table 2:** Aggregate performance of clinical-only, radiomics-only, and clinical–radiomics models.

Model Group	Mean AUC	95% CI	Median AUC	Accuracy	F1	Brier
**Clinical-only**	0.560	0.495–0.612	0.574	0.692	0.806	0.205
**Radiomics-only**	0.676	0.670–0.682	0.682	0.431	0.409	0.335
**Clinical-Radiomics**	0.702	0.696–0.707	0.711	0.523	0.544	0.280

**Table 3:** Feature-selection method performance summary.

Method	Model Group	Mean AUC	Median AUC	AUC SD	F1	Accuracy	Brier
**Graph-FS**	Radiomics only	0.658	0.673	0.055	0.488	0.478	0.300
**Boruta**	Radiomics only	0.666	0.673	0.067	0.387	0.415	0.355
**Lasso**	Radiomics only	0.692	0.704	0.061	0.407	0.430	0.339
**RFE-RF**	Radiomics only	0.667	0.668	0.070	0.360	0.402	0.350
**mRMR**	Radiomics only	0.697	0.698	0.045	0.404	0.429	0.332
**Graph-FS**	Clinical–radiomics	0.703	0.713	0.047	0.641	0.593	0.247
**Boruta**	Clinical–radiomics	0.691	0.698	0.064	0.542	0.515	0.290
**Lasso**	Clinical–radiomics	0.707	0.713	0.059	0.525	0.512	0.285
**RFE-RF**	Clinical–radiomics	0.692	0.712	0.074	0.509	0.496	0.290
**mRMR**	Clinical–radiomics	0.715	0.720	0.047	0.503	0.499	0.289

**Table 4:** Cox external validation summary. Aggregate Cox performance across radiomics parameter settings and feature selection methods. The significant log-rank rate is the percentage of model configurations with a log-rank *p*-value < 0.05 after median risk-score stratification in the external validation cohort.

Model Group/Method	Mean External C-Index	Median C-Index	Significant Log-Rank Rate
**Clinical only**	0.567	0.567	0%
**Radiomics only/Lasso**	0.583	0.585	35%
**Radiomics only/mRMR**	0.573	0.585	35%
**Radiomics only/Graph-FS**	0.565	0.574	30%
**Radiomics only/Boruta**	0.572	0.574	35%
**Radiomics only/RFE-RF**	0.567	0.578	35%
**Clinical–Radiomics/Lasso**	0.598	0.606	55%
**Clinical–Radiomics/Boruta**	0.592	0.603	60%
**Clinical–Radiomics/Graph-FS**	0.591	0.602	55%
**Clinical–Radiomics/mRMR**	0.591	0.604	35%
**Clinical–Radiomics/RFE-RF**	0.587	0.605	55%

## Data Availability

Data sharing is subject to institutional and data-use agreements.

## Supplementary Materials

The following supporting information can be downloaded at: https://www.mdpi.com/article/10.3390/life16061027/s1, Figure S1: Workflow of Graph-Based Feature Selection using Connected Components; Figure S2: Radiomics preprocessing pipeline using ColumnTransformer; Figure S3: Sensitivity analysis of feature-selection stability using a fixed feature-set size of 10; Table S1: Radiomics parameter settings and PyRadiomics extraction configuration; Table S2: RobustScore sensitivity analysis across penalty weights; Table S3: Selected feature-set size in the main analysis; Table S4: Selected feature-set size in the fixed-size sensitivity analysis.

## Abbreviations

HNSCC	Head and Neck Squamous Cell Carcinoma
Graph-FS	Graph-Based Feature Selection
AUC	Area Under the ROC Curve
ROC	Receiver Operating Characteristic
SHAP	Shapley Additive Explanations
mRMR	Minimum Redundancy Maximum Relevance
RFE-RF	Recursive Feature Elimination with Random Forest
RF	Random Forest
XGBoost	Extreme Gradient Boosting
CatBoost	Categorical Boosting
Lasso	Least Absolute Shrinkage and Selection Operator
AJCC	American Joint Committee on Cancer
KI	Kuncheva Index
JI	Jaccard Index
CV	Cross-Validation
CT	Computed Tomography
NS/RO/BW	normalization scale/removal outlier/bin width
Brier Score	Proper scoring rule measuring the accuracy of probabilistic predictions
RobustScore	Mean AUC penalized by variability across parameterizations (introduced in this study)
IQR	Interquartile Range
CI	Confidence Interval
IBSI	Image Biomarker Standardization Initiative
