# Supplementary material for: Impact of Radiomics Parameters and Clinical Integration on Prognostication in Head and Neck Squamous Cell Carcinoma: A Multicenter Study
Source: Life (Basel). 2026 Jun 19;16(6):1027. doi: 10.3390/life16061027 (PMC13302689; doi:10.3390/life16061027)
Supplement: Supplementary file 1 [file life-16-01027-s001.zip › life-4260233-supplementary.pdf]

Supplementary Information

Impact of Radiomics Parameters and Clinical Integration on Prognostication in Head and Neck Squamous Cell Carcinoma: A Multicenter Study

Hajar Moradmand[1*], Jason Molitoris [1], Ranee Mehra [2], Lisa Schumaker [2], Erin Allor[3], Daria A. Gaykalova [3,4,5], Lei Ren [1]

1 Department of Radiation Oncology, University of Maryland School of Medicine, Baltimore, MD 21201, USA; jmolitoris@umm.edu

2 Marlene and Stewart Greenebaum Comprehensive Cancer Center, University of Maryland School of Medicine, Baltimore, MD 21201, USA; ranee.mehra@umm.edu (R.M.)

3 Institute for Genome Sciences, University of Maryland School of Medicine, Baltimore, MD 21201, USA

4 Department of Otorhinolaryngology–Head and Neck Surgery, University of Maryland School of Medicine, Baltimore, MD 21201, USA

5 Department of Oncology, Sidney Kimmel Comprehensive Cancer Center, Johns Hopkins University, Baltimore, MD 21201, USA

* Correspondence: hmoradmand@som.umaryland.edu (H.M.); lren@som.umaryland.edu (L.R.); Tel.: +1-202-340-3730 (H.M. & L.R.)

Supplementary Table S1. Radiomics parameter settings and PyRadiomics extraction configuration

Radiomics features were extracted from the primary gross tumor volume using PyRadiomics. The final analysis used 20 parameter configurations defined by normalization scale (NS), outlier removal threshold (RO), and gray-level bin width (BW).

Parameter setting	NS	RO	BW
Rad_ns50_ro3_bw10	50	3	10
Rad_ns50_ro3_bw15	50	3	15
Rad_ns50_ro3_bw20	50	3	20
Rad_ns50_ro3_bw25	50	3	25
Rad_ns50_ro3_bw30	50	3	30
Rad_ns50_ro4_bw10	50	4	10
Rad_ns50_ro4_bw15	50	4	15
Rad_ns50_ro4_bw20	50	4	20
Rad_ns50_ro4_bw25	50	4	25
Rad_ns50_ro4_bw30	50	4	30
Rad_ns100_ro3_bw10	100	3	10
Rad_ns100_ro3_bw15	100	3	15
Rad_ns100_ro3_bw20	100	3	20
Rad_ns100_ro3_bw25	100	3	25
Rad_ns100_ro3_bw30	100	3	30
Rad_ns100_ro4_bw10	100	4	10
Rad_ns100_ro4_bw15	100	4	15
Rad_ns100_ro4_bw20	100	4	20
Rad_ns100_ro4_bw25	100	4	25
Rad_ns100_ro4_bw30	100	4	30

Shared PyRadiomics settings included image types Original, LoG (sigma = 1.0-5.0), Wavelet, Square, SquareRoot, Logarithm, Exponential, Gradient, LBP2D, and LBP3D; feature classes included shape, first-order, GLCM, GLRLM, GLSZM, and GLDM. Images were resampled to 1 x 1 x 1 mm using B-spline interpolation, with padDistance = 10 and voxelArrayShift = 1000.

Feature Selection Methods

Feature selection plays a pivotal role in radiomics by eliminating redundant, irrelevant, or highly correlated features, thereby improving model generalizability and reducing variance(1) . Redundancy arises when multiple features encode overlapping information, often leading to multicollinearity among predictors. This complicates model interpretation, inflates coefficient standard errors, and can undermine predictive reliability. Addressing these issues is essential for constructing robust and interpretable radiomic models.
Feature selection techniques are generally categorized into three main approaches:

- **Filter Methods** evaluate the statistical association between individual features and the target outcome independently of any learning algorithm. These methods are computationally efficient and well-suited for high-dimensional datasets, but they do not capture inter-feature dependencies, which may lead to suboptimal feature subsets in complex tasks.
- **Wrapper Methods** assess subsets of features by iteratively training and testing predictive models. While they typically yield highly discriminative subsets, their iterative nature makes them computationally intensive, especially with large datasets or resource-heavy models.

- **Embedded Methods** incorporate feature selection within the model training process itself. By penalizing or rewarding certain feature contributions, these methods select features most relevant to predictive performance. Although computationally more efficient than wrappers, they are often model-specific, limiting transferability across different algorithms or datasets.

In this study, four widely used approaches—Boruta, Lasso, mRMR, and RFE—were used as benchmarks for comparison with the proposed Graph-Based Feature Selection (Graph-FS) framework.

I. **Boruta Algorithm (Wrapper):** Boruta identifies all relevant features by leveraging feature importance scores from ensemble methods such as Random Forests (2). For each original featureX_i, a shadow feature $\check{X}_i$ is generated by permuting its value, effectively removing any relation to the outcome variable Y. Random Forest is then trained on the combined dataset, producing important scores $I(X_i)$. Statistical tests compare each $I(X_i)$ with the maximum importance with the maximum importance among shadow features $\left(max\{I(\check{X}_i)\}\right)$. If $I(X_i)$ is significantly higher, X_i is deemed important; otherwise, it is considered unimportant. Features with significantly greater importance are retained, while irrelevant features are iteratively eliminated. Mathematically, a feature X_i is considered important if:

$$I(X_i) > \max\left(\{I(\check{X}_i)\}\right) + \delta$$

Where δ is a significance threshold.

II. **Least Absolute Shrinkage and Selection Operator (Lasso, Embedded):**

Lasso introduces an L_1 regularization penalty that encourages sparsity in regression coefficients (3). The optimization problem is expressed as:

$$min_\beta\left\{\frac{1}{2n}\sum_{i=1}^{n}(y_i - \sum_{j=1}^{p} X_{ij}\beta_j)^2 + \lambda\sum_{j=1}^{p}|\beta_j|\right\}$$

where n is the number of samples, p the number of features, y_i the outcome, and λ the tuning parameter. By shrinking many coefficients to zero, Lasso selects only the most relevant features, reducing dimensionality and improving interpretability.

III. **Minimum Redundancy Maximum Relevance (mRMR, Filter):**

mRMR is a filter-based method that selects features with maximal relevance to the target variable and minimal redundancy among themselves (4). It utilizes mutual information $I(X;Y)$, which quantifies the amount of information one random variable provides about another. Maximum Relevance: $\max_{X_i \in S} I(X_i;Y)$, Minimum Redundancy: $min_{X_i,X_j \in S}\, I(X_i;X_j)$

The combined objective balances these criteria:

$$\max_S\left[\frac{1}{|S|}\sum_{X_i \in S} I(X_i;Y) - \frac{1}{|S|^2}\sum_{X_i,X_j \in S} I(X_i;X_j)\right]$$

IV. **Recursive Feature Elimination (RFE, Wrapper):**

RFE iteratively removes the least important features based on model-derived importance scores (5). At each step, a model (e.g., SVM or Random Forest) is trained, features are ranked by$|I(X_i)|$, and the least important feature is eliminated:

$$X_{(t)} = argmin_{X_i}|I(X_i)|.$$

This process continues until a predefined number of features remain, yielding a subset optimized for model performance.

V. **Graph-Based Feature Selection (Graph-FS, Unsupervised):**

Graph-FS represents features as nodes in a weighted graph, with edges encoding pairwise similarity scores derived from statistical measures such as Pearson correlation or mutual information (6, 7). Formally, the similarity between two features X_i and X_j is defined as:

$$S_{ij} = similarity(X_i, X_j)$$

A graph $G = (V, E)$
is then constructed, with vertices $v_i \in V$ corresponding to features and edges $e_{ij} \in E$ connecting pairs of features with similarity above a predefined threshold. In this work, we employed the Pearson correlation coefficient as the similarity matrix: $S_{ij} = \frac{Cov(X_i, X_j)}{\sigma_{X_i} \cdot \sigma_{X_j}}$
where $Cov(X_i, X_j)$ is the covariance between features, and σ (·) denotes the standard deviation. Once the similarity graph is established, clustering algorithms such as Spectral Clustering or Dominant Set Clustering can be applied to identify groups of interdependent features. Unlike traditional clustering methods that require predefining the number of clusters, dominant sets emerge dynamically from the graph structure, providing flexibility for high-dimensional radiomic data with complex correlations.

Feature selection is then achieved by identifying representative nodes within each cluster. Node ranking is based on graph-theoretic centrality measures. For example, degree centrality, $C(X_j) = \sum_{X_k \in neighbors(X_j)} S_{jk}$, which quantifies the direct connectivity of a feature to its neighbors. Eigenvector Centrality captures both direct and indirect influence of a feature within the global graph structure. Connected Components Analysis was further employed as a complementary strategy. In this approach, the similarity graph is partitioned into connected components, subgraphs in which all features are mutually reachable. Within each component, the feature with the highest degree centrality was retained as the representative. This ensures that redundant features are removed while preserving the most informative members of each correlated group (7).

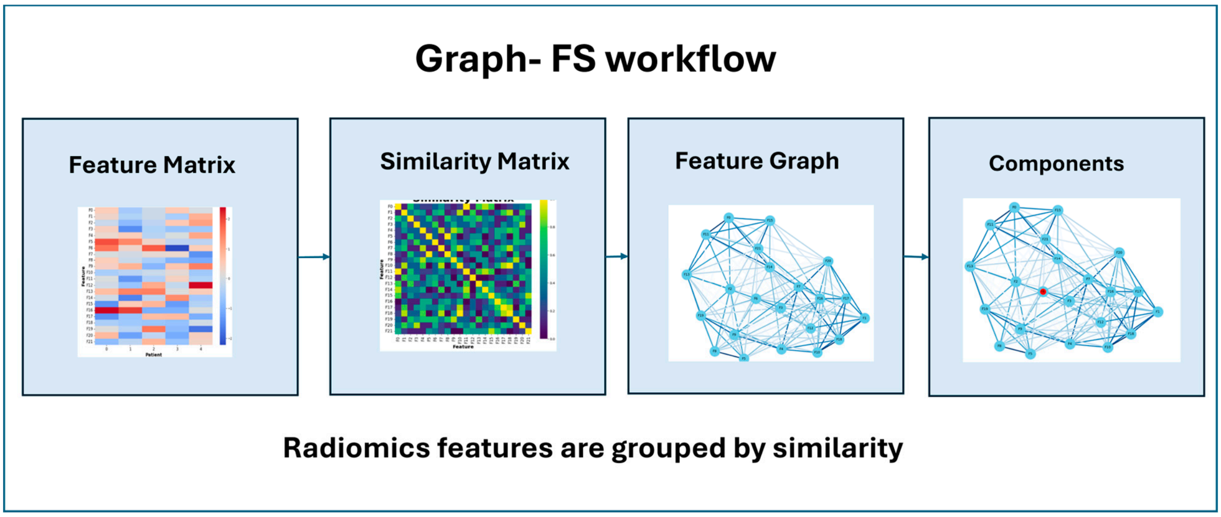

Supplementary Figure S1. Workflow of Graph-Based Feature Selection using Connected Components.

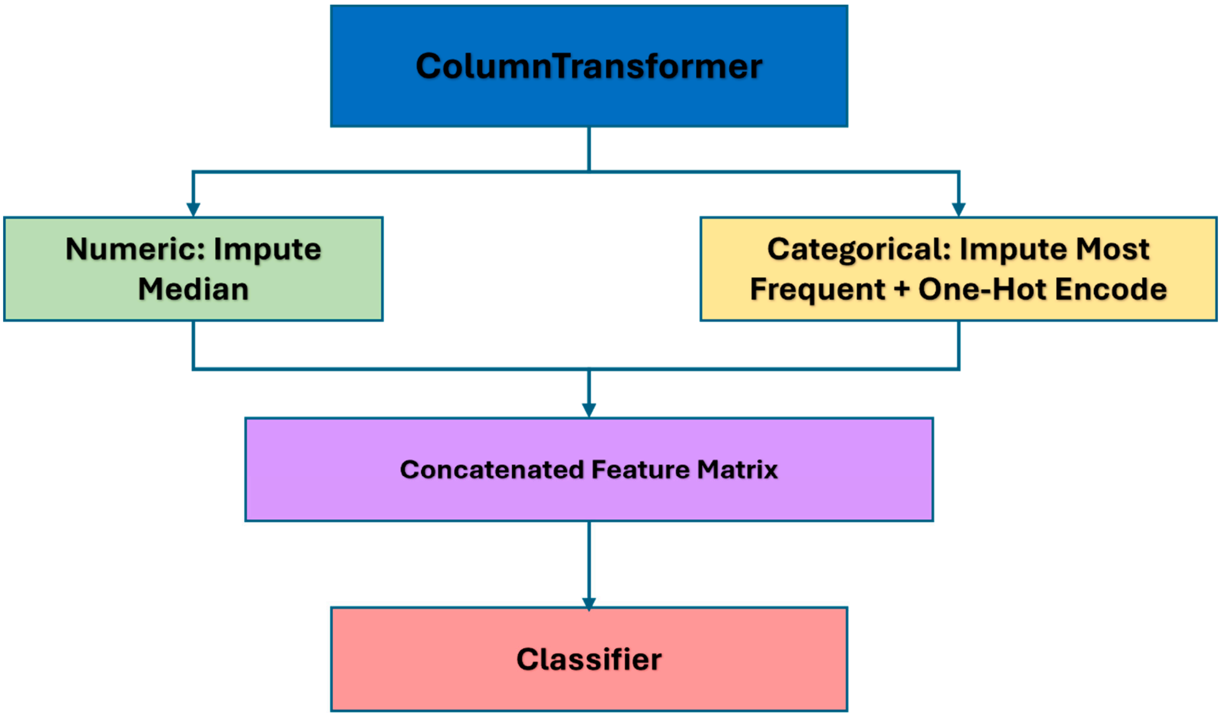

Supplementary Figure S2. Radiomics Preprocessing Pipeline using ColumnTransformer.

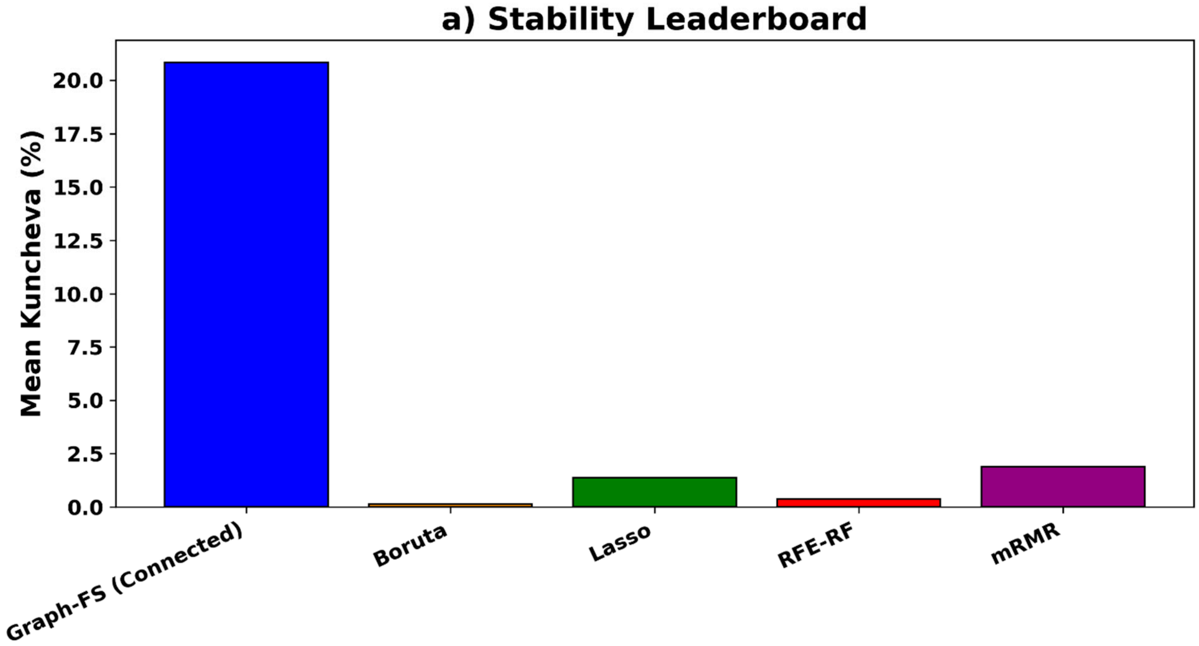

Supplementary Figure S3. Sensitivity analysis of feature-selection stability using a fixed feature-set size of 10.

(a) Stability leaderboard showing the mean Kuncheva index for each feature-selection method when all methods were constrained to select 10 features for every parameter configuration.

Supplementary Table S2. RobustScore sensitivity analysis across penalty weights.

RobustScore was recalculated using lambda = 0.25, 0.50, and 0.75 to assess whether method ranking was sensitive to the penalty applied to AUC variability. The primary analysis used lambda = 0.50.

lambda = 0.25

Method	n runs*	Mean AUC	SD AUC	RobustScore
mRMR	160	0.707	0.046	0.696
Lasso	160	0.699	0.061	0.684
Graph-FS (Connected)	160	0.680	0.057	0.666
RFE-RF	160	0.680	0.072	0.662
Boruta	160	0.676	0.069	0.659

lambda = 0.50

Method	n runs	Mean AUC	SD AUC	RobustScore
mRMR	160	0.707	0.046	0.684
Lasso	160	0.699	0.061	0.669
Graph-FS (Connected)	160	0.680	0.057	0.651
RFE-RF	160	0.680	0.072	0.644
Boruta	160	0.676	0.069	0.641

lambda = 0.75

Method	n runs	Mean AUC	SD AUC	RobustScore
mRMR	160	0.707	0.046	0.673
Lasso	160	0.699	0.061	0.653
Graph-FS (Connected)	160	0.680	0.057	0.637
RFE-RF	160	0.680	0.072	0.626
Boruta	160	0.676	0.069	0.624

* n runs represent the number of classification model configurations per feature-selection method: 20 radiomics parameterizations × 4 classifiers × 2 model groups.

Supplementary Table S3. Selected feature-set size in the main analysis.
The selected feature-set size was summarized across the final radiomics parameter grid used in the main analysis. In this analysis, Graph-FS selected a slightly smaller number of representative features on average than the conventional methods, reflecting its connected-component redundancy-reduction strategy.

Feature selection method	Number of files/configurations	Mean ± SD	Median selected features	IQR	Min–Max
Graph-FS (Connected)	40	8.4 ± 4.8	8.0	4.0–12.0	2–18
Boruta	40	10.0 ± 0.0	10.0	10.0–10.0	10–10
Lasso	40	10.0 ± 0.0	10.0	10.0–10.0	10–10
RFE-RF	40	10.0 ± 0.0	10.0	10.0–10.0	10–10
mRMR	40	10.0 ± 0.0	10.0	10.0–10.0	10–10

Supplementary Table S4. Selected feature-set size in the fixed-size sensitivity analysis.
In the fixed-size sensitivity analysis, the selected feature set size was set to 10 radiomic features across all feature-selection methods and parameter configurations. This analysis was used to evaluate whether differences in feature-selection stability were driven only by unequal subset size.

Feature selection method	Number of files/configurations	Mean ± SD	Median selected features	IQR	Min–Max
Graph-FS (Connected)	40	10.0 ± 0.0	10.0	10.0–10.0	10–10
Boruta	40	10.0 ± 0.0	10.0	10.0–10.0	10–10
Lasso	40	10.0 ± 0.0	10.0	10.0–10.0	10–10
RFE-RF	40	10.0 ± 0.0	10.0	10.0–10.0	10–10
mRMR	40	10.0 ± 0.0	10.0	10.0–10.0	10–10

References

1. Grossberg AJ, Mohamed ASR, Elhalawani H, Bennett WC, Smith KE, Nolan TS, et al. Imaging and clinical data archive for head and neck squamous cell carcinoma patients treated with radiotherapy. Scientific Data. 2018;5(1):180173.
2. Kursa MB, Rudnicki WR. Feature Selection with the Boruta Package. Journal of Statistical Software. 2010;36(11):1 - 13.
3. Tibshirani R. Regression Shrinkage and Selection Via the Lasso. Journal of the Royal Statistical Society: Series B (Methodological). 2018;58(1):267-88.
4. Hanchuan P, Fuhui L, Ding C. Feature selection based on mutual information criteria of max-dependency, max-relevance, and min-redundancy. IEEE Transactions on Pattern Analysis and Machine Intelligence. 2005;27(8):1226-38.
5. Darst BF, Malecki KC, Engelman CD. Using recursive feature elimination in random forest to account for correlated variables in high dimensional data. BMC Genetics. 2018;19(1):65.
6. Ding L, Li C, Jin D, Ding S. Survey of spectral clustering based on graph theory. Pattern Recognition. 2024;151:110366.
7. Moradi P, Rostami M. A graph theoretic approach for unsupervised feature selection. Engineering Applications of Artificial Intelligence. 2015;44:33-45.
